# Comparative genomics of crucian carp-isolated *Flavobacterium psychrophilum*: toward the foundations of overwintering mortality syndrome

**DOI:** 10.3389/fmicb.2026.1751566

**Published:** 2026-02-24

**Authors:** Hucheng Jiang, Zhe Zhao, Yanhua Zhao, Kai Hao, Runbo Li, Aijun Xia, Hui Xue

**Affiliations:** 1 Freshwater Fisheries Research Institute of Jiangsu Province, Nanjing, China; 2 College of Oceanography, Hohai University, Nanjing, China; 3 Jiangsu Marine Fisheries Research Institute, Nantong, China

**Keywords:** comparative genomics, *Flavobacterium psychrophilum*, overwintering mortality syndrome, virulence genes, WGS

## Abstract

*Flavobacterium psychrophilum* is a Gram-negative bacterium that acts as a primary etiological agent of bacterial cold-water disease (BCWD) in salmonid fish. In the past 5 years, this pathogen has also been isolated from multiple freshwater fish species in China, including crucian carp (*Carassius auratus gibelio*), grass carp (*Ctenopharyngodon idellus*) and silver carp (*Hypophthalmichthys molitrix*), and identified as the causal agent of overwintering mortality syndrome. We report the whole-genome sequencing and analysis of *F. psychrophilum* strain NJ01, the first isolate from crucian carp, a cyprinid host. The NJ01 genome consists of a circular chromosome of approximately 2.85 Mb with a GC content of 32.5%, encoding about 2,300 predicted proteins. Comparative genomic analysis with reference strains revealed that NJ01 retains the core genomic features of *F. psychrophilum* while harboring unique genetic elements that may contribute to host specificity and environmental adaptation. NJ01 also harbors genes associated with antibiotic resistance and stress response, suggesting adaptation to aquaculture conditions. Phylogenomic analysis placed NJ01 within a distinct lineage, consistent with CH46 strains isolated from rainbow trout in china. Notably, NJ01 encodes a unique combination of virulence-associated factors such as adhesion proteins, secreted enzymes, and membrane transporters, which may facilitate infection in crucian carp. This study provides novel insights into the genomic basis of *F. psychrophilum* pathogenicity in a cyprinid host. The distinct virulence gene repertoire of NJ01 highlights potential host-specific pathogenic strategies and broadens current understanding of *F. psychrophilum* evolution. These findings may support the development of targeted measures for managing emerging *F. psychrophilum* infections in carp aquaculture.

## 1 Introduction

*Flavobacterium psychrophilum* is the etiological agent of overwintering mortality syndrome in conventional freshwater fish and rainbow trout fry syndrome in salmonid aquaculture (Nematollahi et al., 2003; Jiang et al., 2024), causing substantial losses at low-water temperatures. Although it is primarily associated with trout and salmon, *F. psychrophilum* has also been occasionally isolated from non-salmonid freshwater species, including carp, sturgeon, sea lamprey, eel, and crucian carp (Lehmann et al., 1991; Elsayed et al., 2006; Kumagai and Nawata, 2010). These cases indicate that *F. psychrophilum* is capable of infecting diverse fish hosts, although severe clinical disease in non-salmonids is documented less frequently. Numerous genomic studies of salmonid-derived strains have characterized core and accessory genomic components related to pathogenicity (Duchaud et al., 2007, 2018; Tettelin et al., 2008; Castillo et al., 2016; Rochat et al., 2019). However, all currently published genomes originate from salmonid isolates, leaving a gap in knowledge regarding the genomic characteristics of strains from other hosts.

Crucian carp (*Carassius auratus*) is a widely farmed cyprinid species in Asia (FAO, 2020). Until recently, crucian carp were not recognized as natural hosts for *F. psychrophilum* (Defoirdt, 2014). An outbreak of overwintering mortality syndrome in Jiangsu Province, China, was subsequently attributed to *F. psychrophilum*, marking the first identification of this pathogen in crucian carp (Jiang et al., 2024). These findings raise concerns about an unexpected host-range expansion and the potential for cross-species transmission. Cyprinids differ markedly from salmonids in immune physiology and aquaculture practices, factors that may impose distinct selective pressures on bacterial pathogens. Previous epidemiological studies have suggested that specific *F. psychrophilum* genotypes or clonal complexes tend to associate with particular hosts; for example, certain genotypes predominantly infect rainbow trout, whereas others are linked to non-salmonid species such as ayu or carp (Loman and Pallen, 2015). Such host-specific genotype clustering implies potential adaptive evolution among *F. psychrophilum* lineages.

Despite these observations, the genetic basis of host specificity and virulence variation in *F. psychrophilum* remains poorly understood. Because almost all available genomic data derive from salmonid isolates, the genomic basis for its potential pathogenicity in non-salmonid hosts, such as crucian carp, is entirely unknown. Therefore, characterizing the genome of a crucian carp-associated strain is crucial to identify specific genetic determinants that may contribute to its virulence and host colonization in this novel host (Duchaud et al., 2007, 2018; Tettelin et al., 2008; Castillo et al., 2016; Rochat et al., 2019). However, to definitively elucidate host-adaptive traits, future comparative genomic studies incorporating a diverse collection of strains from both salmonid and non-salmonid hosts will be essential to distinguish genuine adaptive signatures from strain-specific genetic background.

In this study, we report the genome sequence of *F. psychrophilum* strain NJ01, isolated from diseased crucian carp, and present comparative genomic analyses with publicly available *F. psychrophilum* genomes. Specifically, we examined the repertoire of virulence genes, antibiotic resistance genes, stress adaptation factors, and genomic islands in NJ01 relative to reference strains. To our knowledge, NJ01 represents the first genome obtained from a cyprinid host. The objectives were to characterize the NJ01 genome, identify distinctive genetic features, including virulence-associated factors, resistance genes, and mobile elements, and compare them with reference strains to infer potential host-specific adaptations. These findings provide novel insights into *F. psychrophilum* evolution and host-pathogen interactions and support future efforts to manage overwintering mortality syndrome in conventional freshwater fish.

## 2 Materials and methods

### 2.1 Ethics approval

All animal procedures and experimental protocols complied with institutional and national guidelines for the care and use of fish in research and were approved by the Ethics Committee of the Freshwater Fisheries Research Institute of Jiangsu Provincial legislation (Approval Code: FFRI-DW-2023-015). The experimental fish were obtained from the Pukou Base of the Jiangsu Provincial Freshwater Fisheries Research Institute. No antibiotics were used throughout the disease outbreak in the crucian carp pond. To minimize distress and ensure animal welfare during the challenge experiment, fish were anesthetized and handled in an immersion bath containing 50 mg/L ethyl 3-aminobenzoate methane sulfonate.

### 2.2 Bacterial isolation and genome sequencing

*F. psychrophilum* strain NJ01 was isolated from both the surface lesions and liver of a diseased crucian carp collected during an outbreak at a fish farm in Jiangsu Province, China. The affected fish exhibited ulcerative lesions and fin rot consistent with overwintering mortality syndrome. Kidney and spleen tissues were aseptically inoculated onto tryptone yeast extract salt (TYES) agar and incubated at 15 °C for up to 4 days. Yellow-pigmented colonies composed of slender, rod-shaped cells with rounded ends were obtained following isolation. The isolate was identified as *F. psychrophilum* based on Gram staining, biochemical assays, and 16S rRNA gene sequencing, showing 98.67% sequence similarity to the type strain (GenBank accession number: NR_040914.1). A single purified colony was cultured in TYES broth at 15 °C for 48 h with gentle shaking. Genomic DNA was extracted using the TIANamp Bacteria DNA Kit (Catalog No: DP302) following the manufacturer's instructions. Subsequently, DNA quality and concentration were assessed by agarose gel electrophoresis and fluorophotometry using a Qubit 4 instrument. Whole-genome sequencing was performed using both Illumina HiSeq and Oxford Nanopore Technologies (ONT) platforms. Sequencing libraries were constructed separately for each platform, including an Illumina short-read library and an ONT long-read library with 8–10 kb inserts. Raw reads were processed with fastp software (Chen et al., 2018) to remove adapter sequences, trim low-quality bases, and discard reads containing >10% ambiguous nucleotides or < 20 bp after trimming. High-quality clean reads were retained for downstream analyses.

### 2.3 Genome assembly and annotation

*De novo* genome assembly was performed using Unicycler (version 0.4.9) (Wick et al., 2017). High-accuracy Illumina reads (Q30 > 85%) were used to generate an initial contig framework, which was subsequently scaffolded and completed using Oxford Nanopore long reads to obtain the final genome sequence. The assembled genome was polished with Pilon (v1.23) (Walker et al., 2014) to correct sequencing errors. Protein-coding sequences were predicted using Prodigal v2.6.2 (https://github.com/hyattpd/prodigal/wiki), generating annotations for protein-coding genes, total gene length, GC content, and other genomic features. A circular genome map was generated using the R package circlize (Gu et al., 2014) to visualize genomic characteristics.

Secondary metabolite biosynthetic gene clusters were predicted using antiSMASH (v8.0) (Blin et al., 2025). CRISPR arrays were identified using MinCED (v0.4.2). Genomic islands (GIs) were predicted using IslandViewer (v4.0) (Bertelli et al., 2017), and genes within these GIs were compared against CARD (Comprehensive Antibiotic Resistance Database) and VFDB (Virulence Factor Database) to distinguish virulence-associated and resistance genes (Alcock et al., 2023; Zhou et al., 2025). Prophages were predicted using PhiSpy, parameters were set to default (Akhter et al., 2012). Microsatellite sequences (SSRs) were identified using MISA (v2.1), parameters were set to default (Beier et al., 2017). Functional annotation of predicted genes was performed using BLAST (Ye et al., 2006) against the KEGG, GO, and CAZy databases (Kanehisa and Goto, 2000; Consortium, 2003; Cantarel et al., 2009). Perform plasmid analysis using Prokka software (v1.15.6)(Seemann, 2014).

### 2.4 Comparative genomics and phylogenetic analysis

To clarify the phylogenetic context of the NJ01 genome, Representative *F. psychrophilum* genomes were retrieved from NCBI GenBank for comparative analysis, including well-characterized salmonid-derived strains such as JIP02/86 and CSF259–93, as well as strains from diverse hosts and geographical regions (Supplementary Table 1). Core gene families were identified across all genomes using PIRATE, parameters were set to default (Bayliss et al., 2019).

To determine the phylogenetic relationship among *F. psychrophilum* isolates based on genomic data, we selected orthologous genes shared by 20 isolates (2005 genes present in a single copy, paralogs not included) and *F. psychrophilum* NJ01. The corresponding nucleotide sequences were concatenated into a single alignment. A phylogenetic tree was inferred using the Neighbor-Joining algorithm based on pairwise genetic distances calculated with the Kimura 2-parameter model in the MEGA 11 software (Tamura et al., 2011). The robustness of the tree topology was assessed by bootstrap analysis with 1,000 replicates. Bootstrap values of < 80% were removed from the tree. The horizontal bar at the base of the figure represents 0.0005 substitutions per nucleotide site.

The closest relative of NJ01 was identified based on phylogenetic topology and subsequently compared through whole-genome collinearity analysis using Mauve software (Darling et al., 2004). Average nucleotide identity (ANI) values between NJ01 and all referenced *F. psychrophilum* genomes were calculated using the pyani package in Python (Pritchard et al., 2019), and a heatmap was generated to visualize genomic similarities.

### 2.5 Functional annotation and gene enrichment analysis

In order to predict the possible genomic changes in *F. psychrophilum*, the bioinformatics program EDGAR (V3.0) (Dieckmann et al., 2021) was used to predict pan genome of 21 *F. psychrophilum* isolates and calculate the core genome (common genes, mutually conserved) and accessory genome (specific genes, only found in one genome). A Venn diagram was generated with the R package UpSetR (Lex et al., 2014) to visualize the gene distribution patterns. Functional annotation of core and unique genes in the three Chinese *F. psychrophilum* strains NJ01, CH06, and CH46 were performed using the Clusters of Orthologous Genes (COG) database to identify functional differences and metabolic pathway variation.

### 2.6 Identification of virulence genes and antibiotic resistance

Virulence-associated genes unique to or shared among the three Chinese *F. psychrophilum* strains were identified using the VFDB database. Antibiotic resistance genes were detected using CARD, and predicted resistance determinants were classified into six resistance mechanisms. Antimicrobial susceptibility testing was performed exclusively on strain NJ01, as the antimicrobial susceptibility profiles of the other Chinese-origin *F. psychrophilum* strains have been reported in previous studies (Chai et al., 2021). Antimicrobial susceptibility testing was performed using the Kirby-Bauer disk diffusion method (Hudzicki, 2009). Strain NJ01 was cultured on TYES agar medium for 3 days, and 18 antibiotic-impregnated disks were placed aseptically on the culture surface. Following incubation for 18 h, inhibition-zone diameters were measured and visualized using radar charts.

## 3 Results

### 3.1 Genome assembly and general features

**Figure 1:**
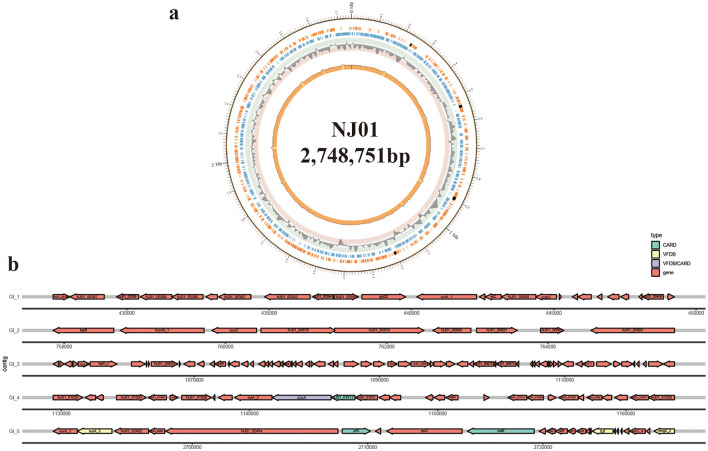
Genome circle map and genomic island (GI) prediction of *F. psychrophilum* NJ01. **(a)** From outside to inside: the first circle represents the genome sequence; the second circle shows coding sequences (CDS) and non-coding RNA regions (rRNA, tRNA) in the reference genome, displayed as outer and inner layers, corresponding to the positive and negative strands, respectively. The third circle shows the GC skew curve calculated using 2,000-bp sliding window, along with the average GC skew, where the dotted line indicates a GC skew of 0. The fourth circle shows the GC content curve calculated using a 2,000-bp sliding window, along with the average GC content of the reference genome, indicated by the dotted line. **(b)** Virulence-associated genes and antibiotic resistance genes present in five GIs were predicted.

The genome assembly and prediction outcomes are summarized in Table 1. The complete genome of *F. psychrophilum* NJ01 was assembled into a single circular chromosome of 2,748,751 bp with a GC content of 32.71%. No plasmids were detected. The genome encodes 2,422 protein-coding sequences, accounting for 87.7% of the genome. Of these, 1,339 (55.3%) were assigned putative functions by database annotation, whereas 1,083 (44.7%) were annotated as hypothetical proteins (Figure 1a). The genome also contains 49 tRNA genes and five rRNA operons; no tmRNA, prophage regions, or CRISPR arrays were identified. Two secondary metabolite biosynthetic gene clusters were detected by antiSMASH, identified as flexirubin and carotenoid, respectively. A total of five GIs were predicted in NJ01, collectively carrying four putative virulence-associated genes (czcA, surA, tuf, mmgC_2) and four antibiotic resistance genes (czcA, NJ01_01011, prfC, rpoB) (Figure 1b). Notably, czcA (a heavy-metal efflux pump) was identified in both categories, suggesting a dual role.

**Table 1:** Genome assembly and prediction results.

**Category**	**Number/copy**	**Total length (bp)**	**% of genome**
Counts of scaffold sequences	1	2,748,751	100
Sequencing depth	560		
Average gene length		969	
GC content in gene region			32.71
Protein-coding genes	2,422	2,411,894	87.74
Intergenic region length		336,857	22.26
tRNA	49	3,694	0.15
rRNA	5	4,144	0.17
tmRNA	0	0	0
CRISPR	0	0	0
GIs	5	165,127	6.84
Prophage	0	0	0
SSR	1364	1,451	0.06
Secondary metabolite region	2	83,004	3.44

### 3.2 Functional annotation of protein-coding genes in strain NJ01

**Figure 2:**
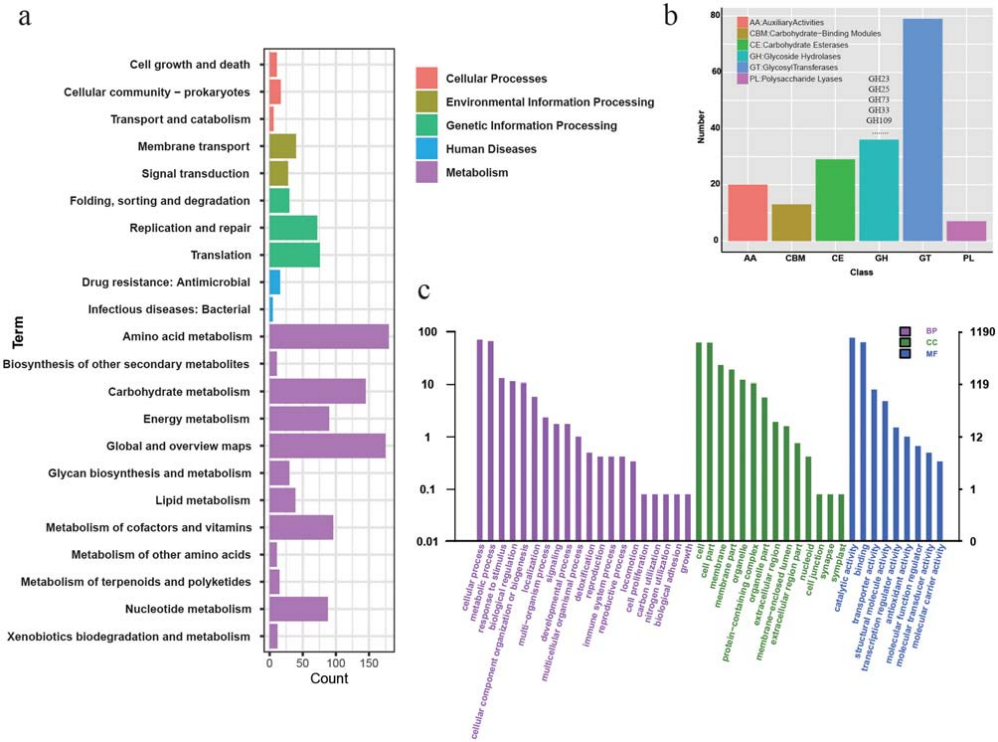
Functional annotation of *F. psychrophilum* NJ01 protein-coding genes using multiple databases. The sequence of each gene was compared against multiple databases to obtain functional annotation. **(a)** KEGG secondary classification of annotated genome sequences. **(b)** Genome sequences annotated to the CAZY database. **(c)** Genome sequences annotated to the GO database. BP, Biological Process; CC, Cell Component; MF, Molecular Function.

Among the 2,422 predicted proteins, 1,193 (49.2%) were annotated with KEGG orthologies, 184 (7.6%) with CAZy families, and 1,190 (49.1%) with GO terms. Of the KEGG-annotated genes, 74.8% (892 genes) were associated with metabolic pathways, 14.9% (178 genes) with genetic information processing (e.g., transcription, replication), and 1.3% (16 genes) with drug resistance (Figure 2a). CAZy analysis showed enrichment in carbohydrate-active enzymes, comprising 79 glycosyl transferases (GTs) and 36 glycoside hydrolases (GHs), including families such as GH23, GH25, GH33, GH73, and GH109 (Figure 2b). GO term analysis indicated that the most represented categories were catalytic activity (76.2%), cellular process (69.7%), and metabolic process (65.5%) (Figure 2c). These annotations suggest that NJ01 possesses a broad metabolic repertoire, consistent with adaptation to diverse substrates in the aquatic environment.

### 3.3 Phylogeny and genome collinearity

**Figure 3:**
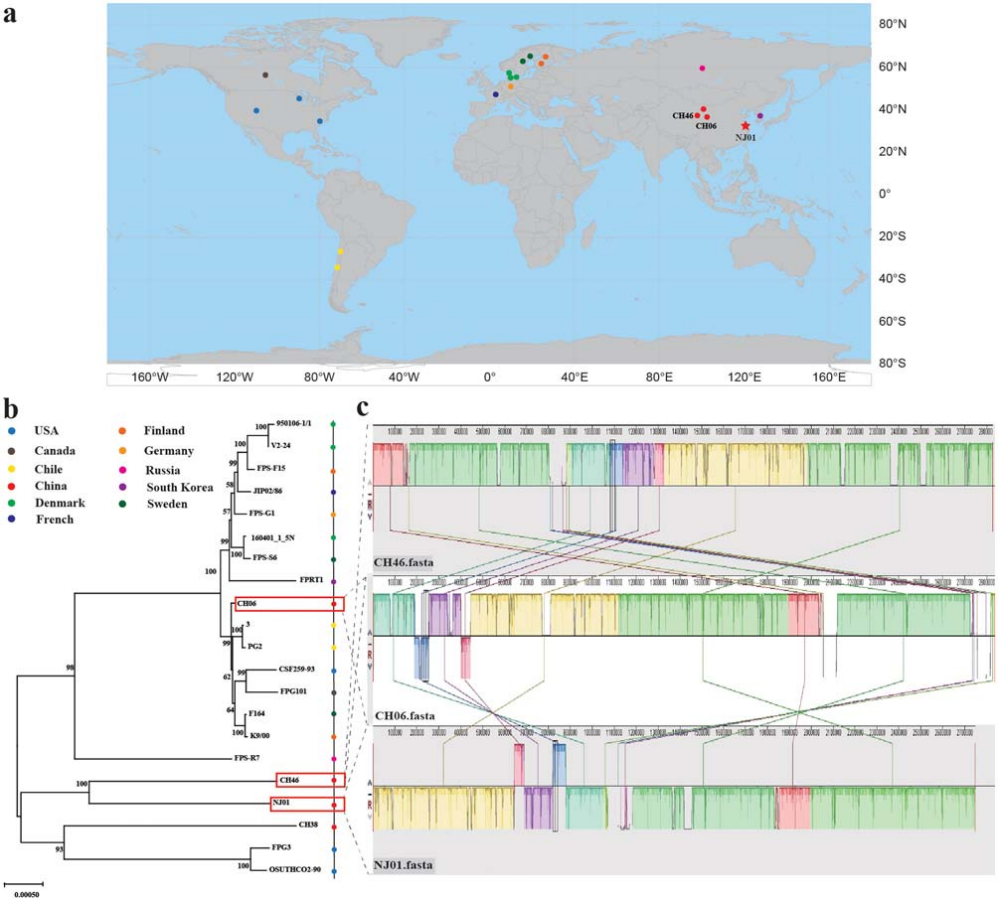
Phylogenomic tree construction and Genome synteny analysis of 21 strains of *F. psychrophilum*. **(a)** Geographical distribution of 21 *F. psychrophilum* strains. **(b)** Core genome phylogeny of *F. psychrophilum isolates*. The maximum likelihood tree was obtained from a concatenated amino acid sequence alignment of the orthologous core genes for the twenty *F. psychrophilum* isolates and the strain NJ01. The numbers above the branches indicate the bootstrap value. Bootstrap values of < 80% were removed from the tree. The horizontal bar at the base of the figure represents 0.0005 substitution per amino acid site. Phylogenetic tree with all strains color-coded according to their country of isolation. **(c)** Genome synteny analysis of three Chinese strains (NJ01, CH06, and CH46). Colors and connecting lines indicate conserved genomic regions.

Phylogenomic tree of 21 *F.psychrophilum* strains based on whole-genome alignment revealed clustering predominantly according to geographic origin (Figure 3a). Several European strains (e.g., FPS_S6, 160401_1_5N, F16, and K900) showed relatively short branch lengths, whereas certain Asian strains, including the Korean strain FPRT1 and the Chinese strains CH38, CH46, and NJ01, displayed longer branches, suggesting greater divergence or a distinct lineage. Among the Chinese isolates, NJ01 clustered most closely with CH46 (Figure 3b). CH06 (a salmonid isolate) also grouped within this clade, whereas CH38 formed a separate branch, highlighting genetic divergence among Chinese strains.

In the phylogenetic tree, CH46 and NJ01 are the most closely related; therefore, we performed synteny analysis on these two strains. Additionally, considering that CH06 represents the first complete *F. psychrophilum* genome reported in China, it was also included in the synteny comparison with NJ01. Genomic synteny analysis demonstrated that NJ01, CH06, and CH46 share extensive conserved syntenic blocks (Figure 3c). The three genomes are largely collinear, with extensive blocks of conserved sequence. Only limited structural variations were observed: CH06 exhibited several small inversions and deletions relative to NJ01 and CH46. These observations indicate that the Chinese strains are highly similar at the genome structural level and likely originate from a common lineage, with NJ01 forming a part of this cluster.

### 3.4 Average nucleotide identity and pangenome analysis

**Figure 4:**
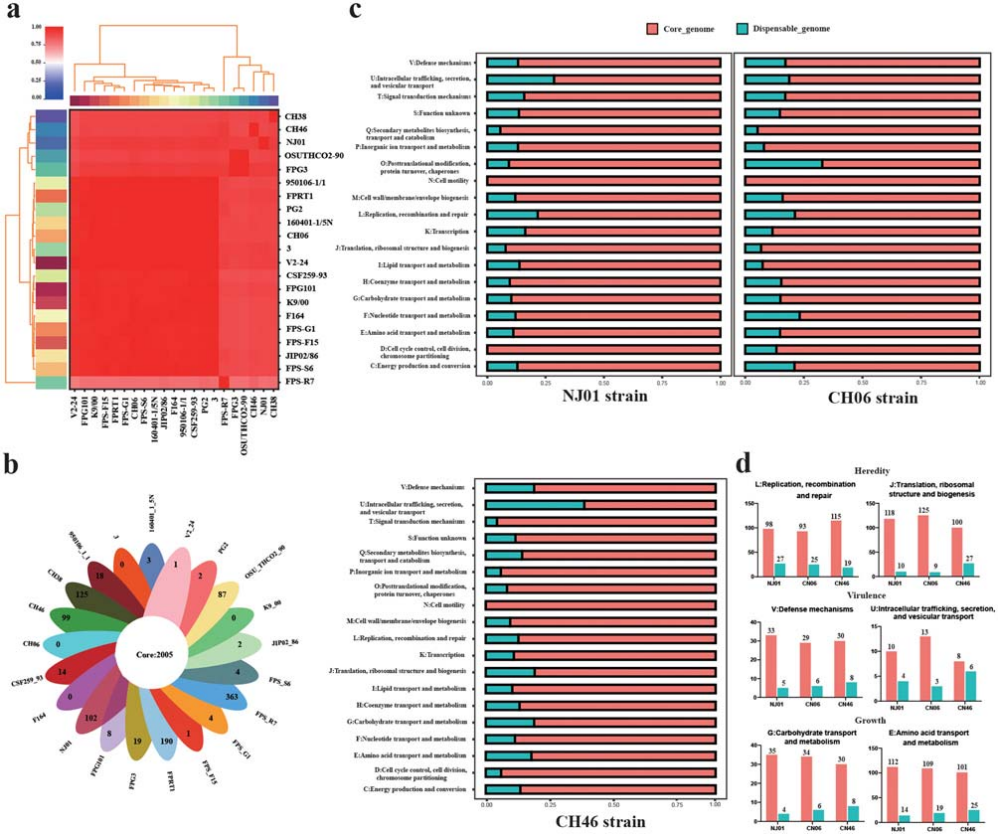
ANI analysis and core genome functional annotation. **(a)** ANI analysis among 21 *F. psychrophilum* strains. **(b)** Venn diagram showing the number of core and unique genes across 21 strains of *F. psychrophilum*. **(c)** COG functional annotation of core and accessory genes in NJ01, CH06, and CH46. **(d)** Comparison of the number of core and accessory genes assigned to genetic, virulence, and growth-related functions in the three strains. The color bars on the left and top of the heatmap share the same meaning: each color represents a distinct cluster group, and strains with the same color exhibit higher similarity at the evolutionary or genomic level. Specifically, the clustering along the top is based on the Neighbor-Joining (NJ) algorithm, while the clustering on the left is performed using the Unweighted Pair Group Method with Arithmetic Mean (UPGMA) algorithm.

Pairwise ANI values between NJ01 and the remaining 20 *F. psychrophilum* genomes ranged from 99.35% to 99.98%, confirming species-level identity. The heatmap of ANI (Figure 4a) showed uniformly high similarity among strains, with the highest values (>99.9%) occurring among geographically proximate isolates. Pangenome analysis of all 21 genomes yielded 2,005 core genes (present in every genome) and 1,037 accessory (strain-specific) genes. The number of unique genes varied by strain: NJ01 possessed 102, CH46 possessed 99, and CH06 harbored comparatively few (not shown) (Figure 4b). Functional characterization of core and accessory genomes of NJ01, CH06, and CH46 showed that core genes were enriched in essential cellular processes, including translation and ribosome structure, cell wall/membrane/envelope biogenesis, and cell motility (Figure 4c). These functions underscore the role of the core genome in fundamental cellular activities. The accessory (non-essential) genome was enriched in functions associated with metabolic pathways and environmental adaptation, including carbohydrate transport and metabolism, amino acid transport and metabolism, and defense mechanisms (Figure 4c). Comparative analysis of genes related to genetic processes, virulence, and growth revealed no significant differences in gene counts across the three strains (Figure 4d). Thus, despite the larger number of unique genes in NJ01, the overall distribution of functional categories remains comparable.

### 3.5 Virulence gene repertoire

**Figure 5:**
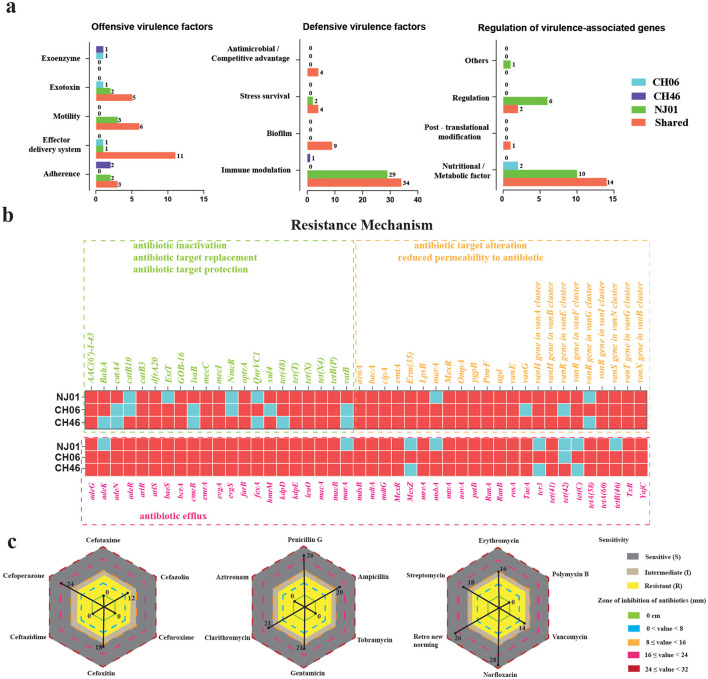
Prediction of virulence and antibiotic resistance genes in three *F. psychrophilum* strains from China. **(a)** Number of common and unique virulence genes in NJ01, CH06, and CH46. **(b)** Prediction of antibiotic resistance genes in three strains according to six categories. Red indicates the presence of resistance genes, and blue indicates the absence of resistance genes. **(c)** Antibiotic susceptibility test results for the NJ01 strain.

A total of 199 candidate virulence genes were identified across NJ01, CH06, and CH46. Among these, 94 genes are shared by all three strains (Figure 5a). These included genes associated with adhesion, exotoxin production, stress response, and immune evasion (Table 2), such as extracellular proteases, hemolysins, type III and type IV secretion systems, and heat-shock proteins.

**Table 2:** Shared virulence factors of NJ01, CH06, and CH46 predicted by VFDB.

**Mechanism**	**Virulence factors category**	**Gene(s)**
Offensive virulence factors	Adherence	htpB, pilR, rpoN
	Invasion	NA
	Effector delivery system	CBU_0270, CBU_1566, CBU_1594, CBU_2076, essC, lirB, ompA, pscN, ricA, vpdC, vscN
	Motility	fleQ, flhF, fliI, flmH, pdxA, ylxH
	Exotoxin	clbD, cyaB, cylB, cylG, hlyB
	Exoenzyme	NA
Defensive virulence factors	Immune modulation	bplF, cap8B, cap8D, cap8E, cap8F, cap8G, Cj1135, gale, gluP, gmd, gtrB, kdsA, kdsB, kdtB, kfiC, kpsF, lgtA, lpxB, lpxD, lpxH, lpxK, msbA, neuA, neuC1, oatA, opsX/rfaC, orfM, pdgA, siaC/synC, tviB, tviC, wbkA, wcaJ, wcbK
	Biofilm	adeG, algB, algC, algI, algR, algU, algZ, mucD, pgaC
	Antimicrobial/Competitive advantage	acrB, farA, farB, mtrD
	Stress survival	clpC, clpP, recN, sodB
Regulation of virulence-associated genes	Post-translational modification	prsA2
	Nutritional/Metabolic factor	basJ, bauE, entA, fepC, iraB, iroN, iutA, mbtN, phzE1, phzH, pvdD, pvdH, shuU, ybtA
	Regulation	phoP, relA
	Others	NA

Each strain also possessed a unique set of virulence-associated genes (Supplementary Table 2). NJ01 harbored 57 unique virulence-associated genes, including *pilH, rfbA*, and *flrB*, which were absent from CH06 and CH46. CH06 contained five unique virulence genes (*basC, clbM, coxFIC1, iucC*, and *speB*), whereas CH46 possessed three (*cpsJ, pilB*, and *pilC*). An additional set of 40 virulence genes present in CH06 and CH46 but absent from NJ01 included 17 genes associated with immune modulation (e.g., *rfbM, cap8M, cpsA/B/E/O, fabZ, manB/C, rffG, lpxC, wzb/c, rpfB, wbcA/G, bplC*), six associated with nutrient acquisition or metabolism (e.g., *fpvA, hitC, mgtB, fbpC, feoB, pvdO*), and five associated with motility (e.g., *motD, cheA/B/Y, fles*). Overall, NJ01 possessed a particularly broad set of virulence factors, with unique genes constituting ~28.6% of its virulence repertoire, compared with CH06 (2.5%) and CH46 (1.5%). This distribution suggests that NJ01 may exhibit a more diverse or aggressive virulence profile, potentially contributing to its pathogenicity toward crucian carp.

### 3.6 Antibiotic resistance genes and susceptibility

The CARD database identified 66 resistance genes across the three strains (NJ01, CH06, and CH46). NJ01 possessed three unique genes (vatB, lsaB, and catA4). CH06 uniquely harbored tet(C), tcr3, mexZ, QnrVC1, and vanR (within the vanG cluster), while CH46 uniquely possessed tet(42), catB10, and NmcR (Table 3). Mechanistic categorization showed that efflux pumps constituted the predominant resistance mechanism, with 44 genes classified in this category (Figure 5b). Three genes were associated with reduced drug permeability, while the remainder encoded machinery for target modification or antibiotic inactivation. In total, 88 gene hits corresponded to resistance against 33 antibiotic types, including penicillins, tetracyclines, sulfonamides, macrolides, cephalosporins, and aminoglycosides.

**Table 3:** Antimicrobial resistance genes of NJ01, CH06, CH46 predicted by CARD.

**Resistance mechanism**	**Antimicrobial resistance genes**	**Drug class**
Antibiotic efflux	mdtA, novA, baeS, kdpD, kdpE	Aminocoumarin antibiotic
	farB	Antibacterial free fatty acids
	adeG, emrA, hmrM, patB, YajC, marA, arlR, arlS	Fluoroquinolone antibiotic
	adeR	Glycylcycline
	adeK, cmeB, evgA/S, macA/B, MexR/Z, mreA, RanA/B, adeN, mtrA	Macrolide antibiotic
	mdsB	Monobactam
	msbA	Nitroimidazole antibiotic
	leuO	Nucleoside antibiotic
	bcrA, rosA, fexA	Peptide antibiotic
	mdtG	Phosphonic acid antibiotic
	TaeA	Pleuromutilin antibiotic
	tcr3, tet (41), tet (42), tet (C), tetA (58), tetA (60), tetB (46), TxR	Tetracycline antibiotic
Antibiotic inactivation	AAC6′)-I-43	Aminoglycoside antibiotic
	NmcR, GOB-16	Carbapenem
	tet (X), tet (X4)	Glycylcycline
	EstT	Macrolide antibiotic
	BahA	Peptide antibiotic
	catA4, catB10, catB3	Phenicol antibiotic
	vatB	Streptogramin antibiotic
	tet (48)	Tetracycline antibiotic
Antibiotic target alteration	vane/G, vanH gene in vanA/B cluster, vanR gene in vane/F/G/I cluster, vanS gene in vanN cluster, vanT gene in vanG cluster, vanX gene in vanB cluster	Glycopeptide antibiotic
	cipA	Lincosamide antibiotic
	MexR, emtA, Erm (35)	Macrolide antibiotic
	arnA, bacA, pgpB, PmrF, ugd	Peptide antibiotic
Antibiotic target replacement	dfrA20	Diaminopyrimidine antibiotic
	mecC, mecI	Penam
	sul4	Sulfonamide antibiotic
Antibiotic target protection	tet (T), tetB (P)	Tetracycline antibiotic
	QnrVC1	Fluoroquinolone antibiotic
	lsaB	Lincosamide antibiotic
	optrA	Oxazolidinone antibiotic
Reduced permeability to antibiotics	LpsB, OmpA	Peptide antibiotic
	marA	Fluoroquinolone antibiotic

Furthermore, NJ01 was tested for antibiotic susceptibility to validate these predictions. Phenotypic susceptibility testing showed that NJ01 was sensitive to nine antibiotics, including penicillin G and ampicillin (penicillins), cefoxitin and cefoperazone (cephalosporins), gentamicin (aminoglycoside), clarithromycin (macrolide), norfloxacin (fluoroquinolone), and streptomycin (Figure 5c). NJ01 was resistant to eight antibiotics: four cephalosporins (cefotaxime, cefazolin, cefuroxime, and ceftazidime); tobramycin (aminoglycoside); aztreonam; polymyxin B; and vancomycin, and it exhibited intermediate resistance to erythromycin. These phenotypes broadly aligned with the genomic predictions, particularly the dominance of efflux pump-associated mechanisms.

## 4 Discussion

*F. psychrophilum* is a globally distributed pathogen causing substantial losses in freshwater salmonid aquaculture (Nematollahi et al., 2003), yet reports from non-salmonid hosts remain comparatively limited. In this study, the *F. psychrophilum* strain NJ01 was successfully isolated from overwintering mortality syndrome in conventional freshwater fish in China. Consistent with previous research from our team, experimental infection of crucian carp with NJ01, followed by histopathological analysis and re-isolation of the bacterium (Jiang et al., 2024), fulfilled all postulates of Koch's criteria. These findings confirm *F. psychrophilum* as an etiological agent can infect cyprinids. NJ01 exhibits typical *F. psychrophilum* morphology, and phylogenomic analysis places it firmly within the *F. psychrophilum* clade, closely related to Chinese salmonid isolates (CH06, CH46). To our knowledge, NJ01 represents the first whole-genome sequence of *F. psychrophilum* derived from a cyprinid host. Comparative genomic analysis across hosts and geographic regions provides essential context for assessing pathogenic potential and evolutionary relationships, informing hypotheses concerning host-range expansion.

The NJ01 genome (2.749 Mb; 32.71% GC) lies at the lower end of the species range (2.685–3.209 Mb; 32.4–34.5% GC). Although niche adaptation may involve shifts in genome size and GC content (Dobrindt and Hacker, 2001), broad surveys have not identified a universal correlation between GC content and growth temperature or environment (Khachane et al., 2005; Kimura et al., 2007; Sato and Kimura, 2019; Liu et al., 2020; Shen et al., 2021; Meyer, 2021; Hu et al., 2022). The slightly reduced GC content observed in NJ01 rRNA genes is notable but cannot be attributed to host-specific adaptation without further evidence.

The genomic analysis of strain NJ01 revealed a highly specialized repertoire of CAZy, a profile consistent with other pathogenic *Flavobacterium* species infecting aquatic animals (Hahnke et al., 2015). Notably, genes encoding peptidoglycan hydrolases/lysozymes (GH23, GH25) and mucin-degrading enzymes (GH33, GH109) were identified within this CAZy profile. These enzymatic functions suggest a dual pathogenic strategy: peptidoglycan hydrolases may facilitate niche establishment by eliminating competing bacteria at infection sites, while mucin-degrading enzymes likely contribute to barrier breaching by degrading the protective mucus layer on host surfaces and visceral tissues. Together, these genomic features provide compelling evidence for the adaptive virulence of NJ01 as a pathogen of crucian carp. Further functional studies are needed to elucidate the specific roles of these key genes during the infection process.

It is noteworthy that, in contrast to many bacteria with abundant secondary metabolites, NJ01 encodes only two conserved pigment biosynthetic gene clusters, a genomic trait consistent with other members of the genus *Flavobacterium* (Silva et al., 2023). This relatively limited repertoire of natural product biosynthesis likely reflects an ecological adaptation strategy of NJ01 as an obligate fish pathogen, wherein its survival depends more on host-derived nutrients and efficient degradative enzyme systems rather than on the synthesis of complex antimicrobial compounds for environmental competition. Previous studies have indicated that flexirubin and carotenoid possess antioxidant properties and contribute to stress resistance (Chhetri et al., 2021). Although the specific roles of these two pigment clusters in *F. psychrophilum* remain unclear, future investigations employing genetic knockouts could further elucidate their precise functions in the pathogenesis of NJ01.

Pan-genome analysis supports a conserved core genome accompanied by a substantial accessory component. Previous studies identified highly similar core genomes at the nucleotide level (Duchaud et al., 2018), documented 1,866 core genes across 26 strains with characteristic COG distributions (Castillo et al., 2016), and described elastin-degrading enzyme FP0506 (JIP02/86) as a non-essential virulence factor distributed variably among 21 strains (Rochat et al., 2019). Consistent with these findings, we recovered ~2,005 core genes among 21 strains, displaying similar functional enrichments. Compared with CH06, NJ01 possesses a larger accessory genome and 102 unique genes, many annotated as putative virulence or stress-response factors, suggesting potential contributions to host adaptation or virulence.

Across NJ01, CH06, and CH46, we detected genes encoding extracellular proteases and hemolysins, in line with established *F. psychrophilum* virulence factors (Kapur et al., 1993; Bertolini et al., 1994; Lorenzen et al., 1997; Whitworth et al., 2005; Högfors-Rönnholm and Wiklund, 2010). NJ01 and CH06 share two key genes, *tlyC* (a hemolysin implicated in evasion of phagocytosis) and *speB* (a protease involved in extracellular-matrix degradation), whereas these loci are absent or divergent in CH46; all three strains harbor *hlyB*. For adhesion, multiple type IV pilus (T4P) genes were identified, including NJ01-unique regulator *pilH*, which may influence motility and surface attachment (Craig et al., 2019). NJ01 also carries two putative flagellar biogenesis genes (*flhF, flil*); although *F. psychrophilum* is considered non-flagellated (Madetoja et al., 2000); these loci may mediate an alternative mechanism of motility or attachment. Another NJ01-unique gene, *flrB*, encodes a histidine kinase containing a PAS heme-binding domain (Mukherjee et al., 2024) and, by analogy with *Vibrio alginolyticus*, may regulate motility and adhesion (Luo et al., 2016). Regarding secretion pathways, type I, Sec, Tat, and type IX (PorSS) systems are well-established in this species (Castillo et al., 2016; Chen et al., 2022). In NJ01, additional genes associated with type III and type IV secretion systems were identified, whereas no type VI secretion system genes were detected, consistent with observations that some *Flavobacterium* species harbor type IV secretion systems but lack type VI secretion systems (Kumru et al., 2020). The diversity of secretion-related genes underscores potentially complex virulence strategies requiring structural and functional validation.

NJ01 contains several GIs harboring adaptive genes (Dobrindt et al., 2004). GI4 contains *czcA*, encoding a Co–Zn–Cd efflux pump that may support survival under heavy-metal stress, whereas GI5 contains *tuf* and *surA*, implicated in protein folding, adhesion, and biofilm formation (Chakraborty et al., 2021). Multiple universal stress-response proteins (e.g., Hsp60, AhpC, SodB, and RecN) likely enhance tolerance to oxidative and thermal stress. Across the three Chinese strains, 88 antimicrobial resistance genes were predicted, including 44 efflux pumps, mechanisms commonly associated with decreased susceptibility to fluoroquinolones, tetracyclines, and macrolides (Webber and Piddock, 2003). Some of these systems may resemble SmeABC/SmeDEF efflux pumps described in *Stenotrophomonas maltophilia* (Sanchez et al., 2005; Brooke, 2012). Phenotypically, NJ01 was susceptible to penicillin G, ampicillin, cefoxitin, cefoperazone, gentamicin, clarithromycin, norfloxacin, and streptomycin, while resistant to cefotaxime, cefazolin, cefuroxime, ceftazidime, tobramycin, aztreonam, polymyxin B, and vancomycin; erythromycin showed intermediate activity (Figure 5c). Intermediate erythromycin susceptibility contrasts with reports of erythromycin-sensitive isolates from the USA and UK (Van Vliet et al., 2017; Ngo et al., 2018), whereas ampicillin susceptibility of CH06 aligns with that observed for NJ01 (Chai et al., 2021). These findings reinforce the importance of antimicrobial stewardship: therapeutic decisions should be guided by strain-specific susceptibility testing to reduce selective pressure and limit the emergence of resistance (Sørum, 2005; Gazal et al., 2020). It is noteworthy that upon comparing the identified antibiotic resistance genes with the results of antimicrobial susceptibility testing, we observed discrepancies between some genotypes and phenotypes, such as Aminoglycoside antibiotic (Tobramycin) and Polypeptide antibiotic (Polymyxin B). This discordance may arise because certain resistance mechanisms require the coordinated action of multiple genes, or because experimental conditions fail to fully replicate the functional expression of these genes (Peterson and Kaur, 2018). Furthermore, some genes may serve multiple physiological roles. For instance, antibiotic efflux involving genes like *mdtA* and *baeS*, while capable of expelling antibiotics, may also participate in clearing environmental toxins, conferring resistance to heavy metals, or facilitating biofilm formation (Blanco et al., 2016). Similarly, lipopolysaccharide biosynthesis genes such as *lpsB* can limit drug entry by reducing permeability to antibiotics (Miller, 2016). Therefore, many resistance genes are pleiotropic, simultaneously underpinning two critical survival strategies in bacteria: fundamental physiological adaptation and antibiotic resistance.

This study presents the first whole-genome sequence of *F. psychrophilum* from crucian carp (NJ01), showing its phylogenetic clustering with other Chinese isolates. Comparative genomics reveals a conserved core together with NJ01-specific genes, including adhesions, secreted enzymes, secretion-system pathways, and antimicrobial-resistance determinants, that likely contribute to cyprinid infection and environmental fitness. Although derived from a single isolate, these findings nominate candidate mechanisms underlying host-range adaptation and highlight targets requiring functional validation. Practically, NJ01′s susceptibility profile may guide overwintering mortality syndrome treatment in conventional freshwater fish. However, it must be clearly stated that this susceptibility data is derived from *in vitro* testing under laboratory conditions. There is currently a lack of systematic research data on the field performance of the corresponding antibiotics against *F. psychrophilum* in complex aquaculture environments, as well as their ecological safety and residue risks. Therefore, any potential application of antibiotics must strictly comply with national regulations for aquaculture medication, be guided by them, and rigorously avoid misuse to ensure aquatic product safety and environmental sustainability. It is worth emphasizing that with the transition of aquaculture toward green and sustainable development, control strategies for *F. psychrophilum* are no longer limited to chemical agents. More targeted and environmentally friendly biocontrol technologies, such as phage therapy (Donati et al., 2021), have made significant progress and demonstrate considerable application potential. Study by Cialini et al. (2025) have shown that the phage therapy can effectively reduce the risk of *F. psychrophilum* invasion by specifically recognizing and lysing. In the future, exploring green and integrated control systems is expected to represent a safer, more efficient, and fundamental direction for controlling this disease. Overall, this study provides a foundation for elucidating *F. psychrophilum* host–pathogen interactions and supporting the development of targeted prevention and control strategies against overwintering syndrome in conventional freshwater fish.

## Data Availability

The complete genome sequence of NJ01 has been deposited to the NCBI (https://www.ncbi.nlm.nih.gov), under BioSample SAMN39450384 and BioProject PRJNA1065097.
