# Supplementary material for: Comparative genomics of crucian carp-isolated Flavobacterium psychrophilum: toward the foundations of overwintering mortality syndrome
Source: Front Microbiol. 2026 Feb 24;17:1751566. doi: 10.3389/fmicb.2026.1751566 (PMC12974136; doi:10.3389/fmicb.2026.1751566)
Supplement: Supplementary file 1 [file Table_1.docx]

**SUPPLEMENTAL MATERIAL**

**Genome of a crucian carp–derived *Flavobacterium psychrophilum* reveals host-specific virulence and adaptive traits**

Hucheng Jiang ^1^, Zhe Zhao^2,3^, Yanhua Zhao^1^, Kai Hao^2,3^, Runbo Li^4^, Hui Xue^1*^, Aijun Xia^1*^

^1^ Freshwater Fisheries Research Institute of Jiangsu Province, Nanjing 210017, China.

^2^ College of Oceanography, Hohai University, Nanjing 210024, China.

^3^ Jiangsu Province Engineering Research Center for Marine Bio-resources Sustainable Utilization, Nanjing 210024, China.

^4^ Marine Fisheries Research Institute of Jiangsu Province, NanTong 226007, China.

***Corresponding author**

Aijun Xia: xia1966@citiz.net

Hui Xue: [jsxuehui@163.com](mailto:jsxuehui@163.com)**Table S1. Twenty strains of *F.psychrophilum* isolated from China and different countries**

| **Strains** | **Host** | **Country** | **Year** | **Size/Mb** | **GC**  **content%** | **GenBank**  **Accession number** |
| --- | --- | --- | --- | --- | --- | --- |
| V2-24 | \ | Denmark | 2014 | 2,684,585 | 32 | CP008881.1 |
| 160401-1/5N | *O.mykiss* | Denmark | 2016 | 2,827,614 | 32.5 | CP039120.2 |
| CH06 | *O.mykiss* | China | 2019 | 2,836,981 | 32.5 | CP046374.1 |
| FPG3 | *O.kisutch* | USA | 2014 | 2,715,909 | 32.5 | CP007207.1 |
| FPS-R7 | *O.mykiss* | Russia | 2017 | 3,208,574 | 32.5 | CP059075.1 |
| CSF259-93 | *O.mykiss* | USA | 1993 | 2,900,735 | 32 | CP007627.1 |
| 950106-1/1 | \ | Denmark | 1995 | 2,839,750 | 32.5 | CP059135.1 |
| CH38 | *O.mykiss* | China | 2020 | 2,830,982 | 32.5 | CP081494.1 |
| CH46 | *T.a.grubei* | China | 2020 | 2,826,602 | 32.5 | CP081493.1 |
| JIP02/86 | \ | French | 2013 | 2,860,382 | 32.5 | AM398681.2 |
| FPG101 | *O.mykiss* | Canada | 2008 | 2,835,130 | 32.5 | CP007206.1 |
| PG2 | *O.mykiss* | Chile | 2009 | 2,850,726 | 32.5 | CP010276.2 |
| F164 | *O.mykiss* | Swedan | 1996 | 2,860,498 | 32.5 | CP059077.1 |
| K9/00 | *O.mykiss* | Finland | 2000 | 2860494 | 32.5 | CP059104.1 |
| FPRT1 | *O.mykiss* | South Korea | 2018 | 2795347 | 32.6 | CP059061.1 |
| FPS-G1 | *O.mykiss* | Germany | 2017 | 2,860,500 | 32.5 | CP059076.1 |
| FPS-S6 | *O.mykiss* | Swedan | 2017 | 2,860,423 | 32.5 | CP039119.2 |
| FPS-F15 | *O.mykiss* | Finland | 2017 | 2,860,487 | 32.5 | CP059093.1 |
| OSU THCO2-90 | *O.kisutch* | USA | 2016 | 2,783,852 | 32.5 | LT670843.1 |
| 3 | \ | Chile | 2014 | 2,805,502 | 32.5 | CP010278.2 |

**Table S2. Unique virulence factors of NJ01, CH06, and CH46 predicted by VFDB**

| **Strain** | **Virulence factors category** | **Gene** |
| --- | --- | --- |
| NJ01 | Adherence | TFP, EF-Tu |
|  | Effector delivery system | LPG_RS00200 |
|  | Immune modulation | cpsA/uppS, cpsB/cdsA, ddrA, lpxD2, lpxM, manB/yhxB, pgi, pks1, rfbA, rfbC, rfbD, rpe, wbpE, wbtD, wbtL, wbtM, wbuZ, GBS_RS06565, GBS_RS06590, hisH2, KP1_RS17330, KP1_RS17335, LPG_RS03745, LPG_RS03830, LPG_RS03850, YE_RS15420, YE_RS15435, ACICU_RS00475, YE_RS15445 |
|  | Motility | flrB, AHML_RS07540, LPG_RS14985 |
|  | Exotoxin | cesB, cesC |
|  | Exoenzyme | tlyC |
|  | Nutritional/Metabolic factor | BioA, bioB, bioF, carA, carB, ggt, panC, panD, purCD, pyrB |
|  | Regulation | bvgA, bvgS, bvrR, letS, mprA, sigA/rpoV |
|  | Stress survival | ahpC, msrA/B |
|  | Others | aatC |
| CH06 | Nutritional/Metabolic factor | basC, iucC |
|  | Effector delivery system | coxFIC1 |
|  | Exotoxin | clbM |
|  | Exoenzyme | speB |
| CH46 | Adherence | pilB, pilC |
|  | Immune modulation | ccpsJ |
| NJ01 & CH06 | NA | NA |
| NJ01 & CH46 | NA | NA |
| CH06 & CH46 | Biofilm | icaR |
|  | Effector delivery system | tagT, eccA1, mycP5 |
|  | Exotoxin | clbJ, clbK, acpC,cylA |
|  | Immune modulation | rfbM, cap8M, cpsO, cpsE, cpsB, cpsA, fabZ, yhxB/manB, rffG, lpxC, wzc, wzb, wzt, manC, rfpB, wbcG, wbcA, bplC |
|  | Motility | motD, cheY, cheB, fleS/flrB, cheA |
|  | Nutritional/Metabolic factor | fpvA, hitC, mgtB, fbpC, feoB, pvdO |
|  | Regulation | bfmR |
|  | Stress survival | katA, msrA/BpilB |
